# A Single-Group Study on the Effect of OnabotulinumtoxinA in Patients with Chronic Migraine Associated with Medication Overuse Headache: Pain Catastrophizing Plays a Role

**DOI:** 10.3390/toxins15020086

**Published:** 2023-01-17

**Authors:** Licia Grazzi, Danilo Antonio Montisano, Paul Rizzoli, Erika Guastafierro, Alessia Marcassoli, Arianna Fornari, Alberto Raggi

**Affiliations:** 1 Centro Cefalee, Fondazione IRRCS Istituto Neurologico Carlo Besta, 20133 Milano, Italy; 2 Brigham & Women’s Faulkner Hospital, John Graham Headacche Center, Harvard Medical School, Boston, MA 02115, USA; 3 UOC Neurologia Salute Pubblica e Disabilità, Fondazione IRRCS Istituto Neurologico Carlo Besta, 20133 Milano, Italy

**Keywords:** OnabotulinumtoxinA, chronic migraine, medication overuse headache, pain catastrophizing, cutaneous allodynia, PREEMPT

## Abstract

Pain catastrophizing and cutaneous allodynia are commonly altered in patients with chronic migraine associated with medication overuse headache (CM-MOH) and tend to improve in parallel with clinical improvement. The relation between pain catastrophizing and cutaneous allodynia is poorly understood in patients with CM-MOH receiving OnabotulinumtoxinA therapy. In this single-arm open-label longitudinal observational study, patients with CM-MOH were assigned to structured withdrawal and then administered OnabotulinumtoxinA (5 sessions on a three-month basis, 195 UI per 31 sites). Headache frequency, medication intake, disability, impact, cutaneous allodynia and pain catastrophizing were evaluated with specific questionnaires. In total, 96 patients were enrolled and 79 completed the 12-month follow-up. With the exclusion of cutaneous allodynia and the magnification subscale of the pain catastrophizing questionnaire, all variables showed significant improvement by the sixth month, which was maintained at 12 months. Reduction of pain catastrophizing, and particularly of its helplessness subscale, was a significant predictor of reduction in headache frequency and medication intake. Pain catastrophizing is often implicated in the clinical improvement in patients with CM-MOH receiving behavioral treatments, but, in this study, also showed a role in patients receiving OnabotulinumtoxinA; combining OnabotulinumtoxinA and behavioral treatments specifically addressing pain catastrophizing might further enhance patients’ clinical outcome.

## 1. Introduction

Chronic migraine (CM) is a primary headache disorder, a progression of episodic migraine (EM), that is characterized by a headache frequency of fifteen or more headache days/month, of whom at least eight per month exhibit typical migraine features, for at least 3 consecutive months [1]. It is often associated with the overuse of symptomatic drugs and, as such, can be seen as a comorbidity for a secondary headache disorder, medication overuse headache (MOH) [1]. MOH is itself characterized by 15 or more headaches/month for more than 3 months: it develops as a consequence of regular medication overuse, and it usually resolves once the overuse is stopped. Specific criteria exist for assigning the MOH diagnosis on the basis of the kind of overused drugs: basically, 15 or more analgesics per month, or 10 or more of the other drugs used to treat migraine, for more than 3 months. The prevalence of MOH ranges between 1% and 2% in the general population [2] and it is associated with poor quality of life (QoL), significant disability, and increased societal burden and cost [3,4,5,6].

As CM-MOH is a condition in which clear biological, psychological and social components are jointly present—i.e., a condition with a biopsychosocial etiology [7]—its treatment should account for each of them, and is therefore based upon three main pillars: (1) withdrawal of overused drugs and prescription of tailored prophylaxis, (2) management of psychological triggers, such as stress, if not direct treatment of mental health aspects, such as anxiety and depression, and (3) education on lifestyle issues (e.g., diet, sleep, physical exercise) [8,9].

Migraine-specific prophylaxis may involve both pharmacological and non-pharmacological treatments, the latter basically including neuromodulation, nutraceuticals and behavioral approaches [10]. Among pharmacological prophylaxis agents, many medications are available, including antidepressants, anti-epileptics, anti-hypertensives, OnabotulinumtoxinA and the newly marketed monoclonal antibodies [11,12,13]. OnabotulinumtoxinA is among the most well-tolerated and safest prophylactic treatments for CM [14,15,16,17], with several studies showing efficacy [16,17], and its therapeutic indication is for CM only.

Different hypotheses have been postulated about the mechanism of action of OnabotulinumtoxinA. When OnabotulinumtoxinA is injected, it enters in the cell cytoplasm cleaving the C-terminus of Synaptosomal-Associated Protein, 25 kDa (SNAP-25), which is the target of botulinum neurotoxin type A both in motor nerve and in sensory nerve terminals. This may result in the inhibition in the release of neuropeptides, inflammatory peptides, substance P, glutamate and calcitonin gene-related peptide (CGRP) within the central nervous system, thus playing a therapeutic role in migraine prevention [18,19]. Its efficacy in headaches other than migraine as well as the factors associated to therapeutic success, however, are not completely clear [20,21].

Several factors have been shown to promote migraine “chronification”, or persistent increase, such as metabolic factors, comorbidities, genetic predisposition, lifestyle, medication overuse, and psychological factors [22,23,24,25,26,27,28,29,30]. Among the latter, pain catastrophizing has been postulated as a potential mechanism of action in the progression of migraine frequency [27,28,29,30]. Pain catastrophizing is a common psychological trait among individuals with migraine, detected in around one fourth of the patients [31], and it is defined as a negative anxiety-driven set of emotions in response to anticipated or actual pain. It is characterized by a set cognitive and emotional features in response to pain, including persistent and invasive thoughts about pain, exaggerated worries about pain and its consequences, and perceived helplessness in response to pain [32]. Patients with high-levels of pain catastrophizing also report higher subjective pain experiences [33]; in fact, pain catastrophizing is considered as a risk factor for pain severity, pain-related disability, psychological distress, and improper use of medications such as analgesics [34].

Pain catastrophizing has been shown to have a role in the process of improvement, in particular following behavioral interventions such as mindfulness and acceptance and commitment therapy [30,35,36,37], but also in relation to pharmacological prophylaxis with erenumab and galcanezumab [38,39], as well as in educational programs [40]. On the contrary, relations between catastrophizing and migraine in patients on OnabotulinumtoxinA therapy was not systematically addressed, with a single study showing improvement in catastrophizing over a six-month period in patients with CM-MOH receiving OnabotulinumtoxinA treatment [41].

The relationship between pain catastrophizing and other variables often implicated in pain experience, such as external locus of control (i.e., the idea that individuals have little possibility to control events) and cutaneous allodynia (i.e., the perception of pain in response to typically innocuous stimuli to the skin, such as heat, cold, or pressure), is poorly understood in patients with CM-MOH receiving OnabotulinumtoxinA therapy.

With this study, therefore, we aimed to enhance our understanding of the role of allodynia and pain catastrophizing in particular by addressing their improvement in patients receiving OnabotulinumtoxinA according to the PREEMPT protocol (Phase III REsearch Evaluating Migraine Prophylaxis Therapy) [42], and by exploring their potential predictive effect on the main outcomes used in headache research, namely headache frequency and medication intake.

## 2. Results

A total of 96 patients were included in the study, 85 (88.5%) were females; mean age was 46.9, SD 9.0, and the mean migraine duration was 28.6 years, SD 9.5. With regard to overused drugs, 40 (41.7%) were triptans over-users only, 16 (16.7%) were analgesics over-users only, and the remaining 40 (41.7%) overused multiple drug classes; additionally, 44 (45.8%) patients showed ASC-12 score ≤ 2, indicative of no allodynia; 29 patients (30.2%) reported headache on a daily basis.

Table 1 reports the differences between study completers and drop-outs for all study variables as measured at the baseline evaluation: no significant differences were observed in any of them. None of the patients complained about adverse events throughout the 12 months.

A total of 17 patients dropped-out before the study’s completion, so longitudinal analyses were carried out over the records of 79 patients. Table 2 reports the longitudinal course of headache frequency and of the other clinical variables. Almost all of the variables showed a significant improvement between baseline and six months, which was then maintained up to 12 months, but no further improvements were recorded after the sixth month. The only exceptions to this were ASC-12 and PCS-Magnification, which were basically stable along the three time points.

Table 3 and Table 4 report the results of the linear regression model predicting the reduction in headache frequency and medication intake between baseline and 6 months. The delta in PCS-Helplessness subscale between baseline and 6 months was the only significant predictor in both models, which accounted for 9.7% of headache frequency reduction and for 12.5% of medication intake reduction.

Table 5 reports the results of the linear regression model predicting the reduction in headache frequency between baseline and 12 months. The delta in PCS-Helplessness subscale between baseline and 12 months was the only significant predictor, and the model accounted for 11.6% of headache frequency reduction.

Table 6 reports the results of the linear regression model predicting the reduction in medication intake between baseline and 12 months. The delta in PCS total score between baseline and 12 months was the only significant predictor, and the model accounted for 10.9% of medication intake reduction.

## 3. Discussion

The results of this study showed that over 12 months, patients with CM associated with MOH who received OnabotulinumtoxinA as prophylaxis according to the PREEMPT study [42] underwent a significant reduction in headache frequency and medication intake, as well as in a set of outcomes such as headache severity, disability, impact and pain catastrophizing, but not allodynia.

OnabotulinumtoxinA is one of the most effective and safest among the available preventive treatments for migraine [14,15,16,17] and the results of our study confirm this. Although we did not implement a systematic data collection for adverse events, none of the patients reported events preventing return to normal activities or requiring treatment interruption, such as some of those reported in a pooled analysis on OnabotulinumtoxinA trials, e.g., neck pain, muscle weakness, facial paresis, stiffness, or respiratory problems [43]. In terms of effect, we showed a reduction by 7.9 days per month at six months (95% CI: 5.6 to 10.3 days) which was maintained at 12 months (mean difference 8.0 days per month, 95% CI: 5.6 to 10.3 days). A recent meta-analysis by Lanteri-Minet and colleagues on patients with CM showed a higher average level of improvement, i.e., 10.6 days (95% CI: 9.2 to 12.3) at six months and 10.3 (95% CI: 5.7 to 14.5) at 12 months [44], and a likewise higher decreased in HIT-6. It has to be noted that, however, the features of the patients herein enrolled are likely different from those resulting from such a pooled analysis. Lanteri-Minet and colleagues did not report average scores for baseline variables, but it has to be noted that for some variables our sample was clearly different; in particular, disease duration was significantly longer (29.2 ± 10.9 years in our study; comprised between 2.4 ± 3.5 and 25.7 ± 12.3 in Lanteri-Minet’s review), whereas HIT-6 was likely lower. In fact, the average HIT-6 score of our patients would place them at the sixth rank out of all the studies which reported HIT-6 (19 studies in total): in a sense, the lower degree of reduction might be due to the lower impact at baseline. Finally, it is difficult to compare the drug intake analysis, as we included the total amount of intake, whereas the review from Lanteri-Minet and colleagues addressed days with acute medication intake.

Patients did not report any significant variation in allodynia scores, as measured by ASC-12 questionnaire, throughout the 12 months of the present study. In previous study in which the ASC-12 was used in patients with CM treated with OnabotulinumtoxinA, a significant ASC-12 decrease was instead observed [45]. However, compared to the patients enrolled by Ozarslan and colleagues [45], the group of patients enrolled in our study reported a lower baseline ASC-12 score (4.4 on average in our study, 6.5 on average in the study of Ozarslan and colleagues). In addition, although the majority of patients in the present sample had ASC-12 scores suggestive of cutaneous allodynia (54.2% of the cases), such a percentage is lower compared to that observed in other studies, e.g., 92.5% by Benatto and colleagues [46], 70.5% by Mathew and colleagues [47], 67.7% by Barbanti and colleagues [48] and 62.7% by Buse and colleagues [49].

The role of pain catastrophizing remains an intriguing psychological concern in migraine patients, as well as in those with chronic pain. Pain catastrophizing is strictly related to several pain-related outcomes, including pain expression, disability and mood impairment, as well as with several physiological, cognitive, affective and interpersonal factors associated with pain [34]: thus, improvement in pain catastrophizing is expected to produce improvement in pain severity, and eventually vice versa. Pain catastrophizing is a common facet of migraine, being present in around one-fourth of the patients and associated with migraine attacks severity and migraine chronification, as well as disability, particularly among those with CM [31,50,51,52], and it has been shown to improve following non-pharmacologic interventions [35,37,52,53,54]. However, improvement in pain catastrophizing following pharmacological treatment was, to the best of our knowledge, previously reported in two real-life studies on erenumab and galcanezumab [38,39], and for the first time in the present study following OnabotulinumtoxinA treatment.

Such an improvement in pain catastrophizing is of primary clinical relevance, as it is associated with decreased quality of life, and greater use of healthcare services and longer hospital stays in pain-related syndromes [55,56]. In particular, among migraine sufferers it is associated with higher attacks frequency, poor treatment response, increased medical consultation, higher disability and lower health-related quality of life [57]. The fact that it not only improves after treatment, but that pain catastrophizing improvement was an independent predictor of both the six-month and the twelve-month reduction in headache frequency and medication intake, with the adjusted model accounting for 9.7% to 12.5% of the variance in these outcomes, provides further evidence to the importance of such a dimension. In particular, improvement in the helplessness dimension (i.e., patients’ belief that they can do nothing to alleviate pain), seemed to play a role in determining the observed improvement among our patients. We hypothesize that through the therapy patients gathered confidence and ability in managing both acute treatments’ use, recognizing when they are really needed, and thus limiting the amount of days with headache. Parallel to this, it has to be taken into account the administration modality of OnabotulinumtoxinA: in fact, the three-month periodic meeting with the neurologist, which encompass discussion on the clinical course of migraine (clearly only in real-world settings) clearly plays a role in inducing clinical advantage related to an improvement of mental constructs.

Some limitations need to be acknowledged. First, we did not have a control condition, for example another kind of pharmacological prophylaxis: therefore, we cannot entirely disentangle the effect of the specific treatment with OnabotulinumtoxinA from that of the general clinical improvement. The regression models we applied provide justification on the direction of the relation: however, a strict causal relation explaining headache frequency and medication intake reduction cannot exclude OnabotulinumtoxinA treatment which, being an inclusion criterion, cannot be part of the regression models. Second, it was a monocentric study, with the sample being drawn from a third-level specialty center where patients with a considerable disease severity and long history of CM-MOH are expected to attend. Third, in relation to the previous limitation, it has to be taken into account that clinicians working in our center promote patients’ awareness of their mental states and on the correct use of medication. The degree to which such an approach promoted the aforementioned improvement in pain catastrophizing, which had an independent effect on headache frequency and medication intake reduction, cannot be ascertained: however, it cannot be completely ignored. Finally, a note on the possible effects of imputation on the precision of estimates is needed. In fact, the error term that is produced by the imputation is likely lower than the error that would be produced by “real cases”. The implication of this is a possible bias the relations between different variables: this in turn might produce a degree of precision in the imputed values which is higher than warranted, and the predictive power of regression models might be overestimated. However, we think that, considering the trend observed among real cases (i.e., a wide improvement between baseline and 6 months, and then substantial stability over the next six months) and the fact that we imputed only the 12 month values, the overall results of our study would not be significantly different if attrition was smaller. Therefore, we deem that the overall degree of bias can be acceptable. 

## 4. Conclusions

In conclusion, the results of this study show that headache frequency, medication intake and pain catastrophizing improved in patients receiving OnabotulinumtoxinA prophylaxis for CM-MOH. Such an improvement is evident at six months from the inception of the treatment, and is maintained up to 12 months. Moreover, pain catastrophizing, and particularly its helplessness dimension, was predictive for decreased headache frequency and medication intake over 12 months.

Learning how to manage headache pain is a process that deals with avoiding catastrophizing in relation to pain. It is reasonable to presume that combining OnabotulinumtoxinA prophylaxis with a behavioral treatment specifically addressing pain catastrophizing might further on enhance patients’ clinical outcome.

## 5. Materials and Methods

### 5.1. Study Design and Patients’ Selection Criteria

A longitudinal single-arm open-label retrospective observational study design was implemented. Clinical documentation of patients who met the criteria of the International Classification Headache Disorders, third version (ICHD-3) [1] for Chronic Migraine (CM, code 1.3) with associated Medication Overuse Headache (MOH, code 8.2) and who received OnabotulinumtoxinA as prophylaxis following the PREEMPT protocol [42] were included. Known intolerance to OnabotulinumtoxinA and pregnancy constituted exclusion from the treatment. All patients signed an informed consent form for the standard treatment and a second for the use of retrospective data: the study was retrospectively authorized by the Institute’s Ethical Committee (approval n. 66/2022, 14 September 2022). Patients were enrolled between January 2018 and December 2019.

Patients were enrolled at the time they entered a structured in-hospital withdrawal treatment, which was organized in a day hospital regimen for 5 days in order to stop the overuse of symptomatic medications. The main pillars of structured withdrawal included [58,59]: intravenous hydration, intravenous steroids, oral benzodiazepines, oral or intravenous metoclopramide or indomethacin if needed for intense rebound headache. Overused drugs were abruptly stopped, and tailored counseling on the correct use of medications was provided. As part of patients’ education, recommendations on the consumption of at least three regular meals per day, sleep hygiene (7–8 h of sleep per night) and on physical activity (20–30 min per day of moderate level physical activity) was included. All patients were advised to stop work or any other activity during the withdrawal and to stay at home and rest as much as possible after the therapy.

Once structured withdrawal was completed, patients were administered OnabotulinumtoxinA as prophylaxis following the PREEMPT protocol [42]: 5 sessions on a three-month basis, at the dosage of 195 UI per 31 sites. The PREEMPT protocol is now a standard for treatment of CM-MOH and patients have access to it by co-paying the whole cost, which for approximately 80% is covered by the Italian National Health System. Patients were followed up for one year.

### 5.2. Research Protocol

Monthly headache frequency, measured with headache diaries, was used as primary endpoint. In addition to headache frequency, diaries included a section on the total intake of symptomatic medications. Both headache frequency and medication intake were evaluated at each OnabotulinumtoxinA administration, i.e., every three months. Other collected data included measures of disability and severity, impact, cutaneous allodynia and catastrophizing attitude: these questionnaires were administered at the baseline, at six and at twelve months.

Disability and average headache severity were measured with the Migraine Disability Assessment (MIDAS) [60]. The MIDAS is composed of seven items referred to the preceding 3 months. The first five address the influence of headache in different domains and patients have to refer the number of days in which: they were completely unable to carry out paid and schoolwork activities (item 1) or limited in 50% or more of their ability in the same activities (item 2); completely unable to carry out household work (item 3), or limited in 50% or more of their ability in the same activities (item 4); finally, the fifth item addresses the number of days in which headaches had an impact (full or partial) over leisure activities with family or in social situations. MIDAS score is the sum of responses to questions 1–5, it ranges between 0 and 270, and four severity grades are available: minimal (0–5), mild (6–10), moderate (11–20), and severe (>21) disability. The last two items investigate the total number of days with migraine attacks and the average pain intensity (0–10 scale).

The Headache Impact Test (HIT-6) [61] is a 6-item scale that measures lost time in 3 domains and other areas of impact (e.g., pain severity, fatigue, and mood), based on patient’s recall of the previous past 4 weeks. Each item is rated on a scale ranging from “never” to “always”, and HIT-6 total score ranges between 36 and 78, with higher scores indicating greater impact: scores ≥ 60 are indicative of a severe impact.

The 12-item Allodynia Symptom Checklist (ASC-12) [62] was used to measure cutaneous allodynia. The ASC-12 is composed of items reflecting situations in which increased unpleasant skin sensation or pain can be experienced during a migraine attack (e.g., combing hair, putting eyeglasses on, resting face on a pillow). For each of the 12 items patients have to respond identifying how often they experience such a sensation (never, rarely, less than half of the time, half of the time or more), and they can also reply that the item does not apply (e.g., people who do not wear contact lenses or earrings). Never and rarely are scored as 0, less than half of the time is scored as 1, half of the time or more is cored as 2, so that the total score range is 0–24. ASC-12 score 0–2 indicates no allodynia, 3–5 mild allodynia, 6–8 moderate allodynia and 9 or more indicates severe allodynia.

The Pain Catastrophizing Scale (PCS) [63] was used a measure of catastrophizing as it relates to pain, i.e., an exaggerated negative mental set brought to bear during actual or anticipated painful experience. The PCS is composed of 13 items that form three subscales, which identify the dimensions of rumination (the constant thinking about pain), magnification (the exaggeration of pain and of its consequences), and of helplessness (the belief that there is no or limited possibility that pain may improve). Items are to be rated between 0 and 4 (from “not at all” to “all the time”) and total PCS score ranges between 0 and 52, with higher scores indicating a higher tendency to catastrophize, and scores ≥30 indicating clinically significant levels of catastrophizing.

### 5.3. Data Analysis

Due to the important drop-out rates related to COVID-19 pandemics, we operated a missing value imputation for those patients who were lost at the follow-up between the sixth and the twelfth month due to the important limitations in mobility imposed by the pandemic. In fact, almost half of the study participants could did not attend follow-up (45 out of 96), of whom approximately two-thirds (28 of 45 patients) dropped-out between month six and month twelve during the first waves of COVID-19 pandemics in 2020. The last observation carried forward approach was used for missing data imputation. The rationale for this was that our analysis showed that the variation in the herein used endpoints was reported between baseline and six months and, conversely, no change or only minimal changes were observed between six and twelve months.

We calculated baseline differences between study completers (including those who were imputed) and drop-out using independent-sample t-test, and longitudinal differences using a repeated-measures ANOVA, with Bonferroni post-hoc analysis. Mauchly’s W test of sphericity was used, and Hyun-Feldt or Greenhouse–Geisser corrections were used when sphericity was violated: the Hyun-Feldt correction was used when Mauchly’s W test was higher than 0.75, and the Greenhouse–Geisser when W was lower than 0.75.

Finally, four multivariable linear regression models were implemented to address the predictors of the following: headache frequency change between baseline and six months and between baseline and twelve months; medication intake change between baseline and six months and between baseline and twelve months. Predictors were the respective change, i.e., delta between baseline and six months and delta between baseline and twelve months, in ASC-12, PCS-total and PCS subscales. A backward approach was used, with variables entered together and then excluded if they were not significantly associated to the outcome (i.e., exclusion criterion was *p*-value ≥ 0.05).

Data were analyzed with SPSS 28.0.

## Figures and Tables

**Table 1:** Descriptive sociodemographic and clinical variables: differences between study completers and drop-outs.

	Completers	Drop-Out	t	*p*
Age	46.6 ± 8.2	48.6 ± 12.3	0.65	0.524
Disease duration	29.2 ± 10.9	25.6 ± 12.5	1.12	0.274
Monthly headache frequency	21.9 ± 6.5	20.7 ± 6.4	0.75	0.458
Monthly medication intake	22.0 ± 10.5	27.6 ± 20.4	1.11	0.281
MIDAS	74.4 ± 60.4	77.5 ± 48.6	0.20	0.844
Average headaches severity	7.8 ± 1.3	7.4 ± 1.4	1.01	0.316
HIT-6	65.3 ± 6.7	65.9 ± 5.0	0.35	0.730
ASC-12	4.4 ± 4.4	3.8 ± 3.4	0.47	0.638
PCS total score	30.2 ± 11.2	32.9 ± 10.3	0.91	0.364
PCS-Helplessness	13.8 ± 5.6	14.5 ± 4.7	0.43	0.666
PCS-Rumination	13.8 ± 4.5	15.1 ± 5.1	1.00	0.318
PCS-Magnification	46.6 ± 8.2	48.6 ± 12.3	0.65	0.524
PCS-Magnification	2.6 ± 2.2	3.2 ± 2.2	0.99	0.325

Notes: MIDAS, Migraine Disability Assessment; HIT-6, Six-item Headache Impact Test; ASC-12, Twelve-item Allodynia Symptom Checklist; PCS, Pain Catastrophizing Scale. Age and Disease duration was expressed in years; Monthly headache frequency was expressed in days; Monthly medication intake was expressed in number of single assumptions; Average headaches severity was expressed as 0–10 Visual Analogue Scale.

**Table 2:** Longitudinal course of sociodemographic and clinical variables.

	Mauchly’s W (*p*) ε	Within Subjects’ F (*p*)	Means (95% CI) of the Estimates			Means (95% CI) of the Differences	
			Baseline	6M	12M	Baseline-6M	Baseline-12M
Monthly headache frequency	0.833 (<0.001) ε = 0.874	F(1.7,136.3) = 55.4 (<0.001)	21.9 (20.4; 23.4)	14.0 (12.4; 15.6)	13.9 (12.3; 15.5)	7.9 (5.6; 10.3)	8.0 (5.7; 10.4)
Monthly medication intake	0.823 (<0.001) ε = 0.866	F(1.7,135.2) = 21.6 (<0.001)	22.0 (19.4; 24.3)	14.8 (12.1; 17.5)	14.5 (12.3; 16.7)	7.2 (3.6; 10.8)	7.5 (4.1; 10.9)
MIDAS	0.820 (<0.001) ε = 0.864	F(1.7,134.8) = 11.2 (<0.001)	74.4 (60.9; 88.0)	51.8 (40.5; 63.0)	50.3 (40.6; 59.9)	22.7 (6.7; 38.6)	24.2 (9.5; 38.9)
Average headaches severity	0.893 (0.013) ε = 0.923	F(1.8,144.1) = 15.5 (<0.001)	7.8 (7.5; 8.0)	7.1 (6.8; 7.4)	7.2 (6.9; 7.6)	0.7 (0.4; 1.0)	0.5 (0.2; 0.9)
HIT-6	0.971 (0.321)	F(2,156) = 10.1 (<0.001)	65.3 (63.8; 66.9)	61.9 (60.4; 63.5)	62.5 (61.1; 63.8)	3.4 (1.3; 5.5)	2.9 (0.9; 4.9)
ASC-12	0.939 (0.088)	F(2,156) = 1.1 (0.349)	4.4 (3.4; 5.3)	3.9 (2.8; 4.9)	4.0 (3.0; 4.9)	0.5 (−0.4; 1.4)	0.4 (−0.5; 1.3)
PCS total score	0.927 (0.053)	F(2,156) = 18.8 (<0.001)	30.2 (27.7; 32.7)	24.6 (22.2; 27.1)	25.8 (23.3; 28.4)	5.6 (3.4; 7.7)	4.4 (1.7; 7.0)
PCS Helplessness	0.883 (0.008) ε = 0.915	F(1.8,142.8) = 23.6 (<0.001)	13.8 (12.6; 15.1)	10.6 (9.4; 11.9)	11.1 (9.9; 12.4)	3.2 (2.0; 4.4)	2.7 (1.3; 4.1)
PCS Rumination	0.960 (0.205)	F(2,156) = 13.0 (<0.001)	13.8 (12.8; 14.8)	11.8 (10.8; 12.9)	12.3 (11.3; 13.4)	2.0 (0.9; 3.0)	1.5 (0.4; 2.5)
PCS Magnification	0.980 (0.456)	F(2,156) = 1.4 (0.241)	2.6 (2.1; 3.1)	2.3 (1.8; 2.8)	2.4 (1.9; 2.8)	0.3 (−0.2; 0.8)	0.3 (−0.2; 0.8)

Notes. All pairwise comparison between baseline and 6 months’ follow-up and between baseline and 12 months’ follow-up show significant differences except for ASC-12 and PCS-Magnification. None of the pairwise comparison between 6 months’ and 12 months’ follow-up show significant differences (data not shown). For Monthly headache frequency, Monthly medication intake, MIDAS, Average headaches severity, and PCS-Helplessness, the Hyun-Feldt ε correction was used due to sphericity violation. MIDAS, Migraine Disability Assessment; HIT-6, 6-item Headache Impact Test; ASC-12, 12-item Allodynia Symptom Checklist; PCS, Pain Catastrophizing Scale. Monthly headache frequency was expressed in days; Monthly medication intake was expressed in number of single assumptions; Average headaches severity was expressed as 0–10 Visual Analogue Scale.

**Table 3:** Regression model predictive of headache frequency variation between baseline and the six-month follow-up.

	Multivariable Regression: Initial Model		Multivariable Regression: Final Model	
	Beta	*p*	Beta	*p*
Constant	-	<0.001	-	<0.001
Δ (0–6M) ASC-12	0.146	0.201		
Δ (0–6M) PCS total score	−0.364	0.673		
Δ (0–6M) PCS-Helplessness	0.491	0.300	0.330	0.003
Δ (0–6M) PCS-Rumination	0.160	0.729		
Δ (0–6M) PCS-Magnification	0.146	0.416		
Model fit	R^2^ = 0.139; Adj R^2^ = 0.080 F = 2.36 (*p* = 0.048)		R^2^ = 0.109; Adj R^2^ = 0.097 F = 9.39 (*p* = 0.003)	

Notes. ASC-12, 12-item Allodynia Symptom Checklist; PCS, Pain Catastrophizing Scale.

**Table 4:** Regression model predictive of medication intake variation between baseline and the six-month follow-up.

	Multivariable Regression: Initial Model		Multivariable Regression: Final Model	
	Beta	*p*	Beta	*p*
Constant	-	0.032	-	<0.001
Δ (0–6M) ASC-12	0.030	0.792		
Δ (0–6M) PCS total score	−0.198	0.817		
Δ (0–6M) PCS-Helplessness	0.539	0.253	0.369	<0.001
Δ (0–6M) PCS-Rumination	0.004	0.993		
Δ (0–6M) PCS-Magnification	−0.017	0.924		
Model fit	R^2^ = 0.146; Adj R^2^ = 0.088 F = 2.50 (*p* = 0.038)		R^2^ = 0.136; Adj R^2^ = 0.125 F = 12.15 (*p* < 0.001)	

Notes. ASC-12, 12-item Allodynia Symptom Checklist; PCS, Pain Catastrophizing Scale.

**Table 5:** Regression model predictive of headache frequency variation between baseline and the 12-month follow-up.

	Multivariable Regression: Initial Model		Multivariable Regression: Final Model	
	Beta	*p*	Beta	*p*
Constant	-	<0.001	-	<0.001
Δ (0–6M) ASC-12	0.111	0.314		
Δ (0–6M) PCS total score	−0.437	0.268		
Δ (0–6M) PCS-Helplessness	0.628	0.021	0.357	0.001
Δ (0–6M) PCS-Rumination	0.071	0.762		
Δ (0–6M) PCS-Magnification	0.153	0.232		
Model fit	R^2^ = 0.164; Adj R^2^ = 0.107 F = 2.86 (*p* = 0.021)		R^2^ = 0.127; Adj R^2^ = 0.116 F = 11.25 (*p* = 0.001)	

Notes. ASC-12, 12-item Allodynia Symptom Checklist; PCS, Pain Catastrophizing Scale.

**Table 6:** Regression model predictive of medication intake variation between baseline and the 12-month follow-up.

	Multivariable Regression: Initial Model		Multivariable Regression: Final Model	
	Beta	*p*	Beta	*p*
Constant	-	<0.001	-	<0.001
Δ (0–6M) ASC-12	−0.013	0.906		
Δ (0–6M) PCS total score	0.527	0.188	0.347	0.002
Δ (0–6M) PCS-Helplessness	0.064	0.813		
Δ (0–6M) PCS-Rumination	−0.203	0.393		
Δ (0–6M) PCS-Magnification	−0.117	0.365		
Model fit	R^2^ = 0.143; Adj R^2^ = 0.085 F = 2.42 (*p* = 0.042)		R^2^ = 0.121; Adj R^2^ = 0.109 F = 10.56 (*p* = 0.002)	

Notes. ASC-12, 12-item Allodynia Symptom Checklist; PCS, Pain Catastrophizing Scale.

## Data Availability

The dataset used to prepare the analyses included in this manuscript have been archived on Zenodo (https://doi.org/10.5281/zenodo.7405322), accessed on 15 January 2023.
